# The introduction of an intestinal ultrasound service is associated with significantly reduced endoscopy usage in an inflammatory bowel disease service—the SCOPELESS study

**DOI:** 10.1093/ecco-jcc/jjag068

**Published:** 2026-05-28

**Authors:** Grace S Feng, Robert D Little, Zaid Ardalan, Antony B Friedman, Rebecca L Smith, Kirstin M Taylor, Miles P Sparrow

**Affiliations:** Department of Gastroenterology, Alfred Health, Melbourne, Australia; Department of Gastroenterology, Alfred Health, Melbourne, Australia; School of Translational Medicine, Monash University, Melbourne, Australia; Department of Gastroenterology, Alfred Health, Melbourne, Australia; School of Translational Medicine, Monash University, Melbourne, Australia; Department of Gastroenterology, Alfred Health, Melbourne, Australia; School of Translational Medicine, Monash University, Melbourne, Australia; Department of Gastroenterology, Alfred Health, Melbourne, Australia; School of Translational Medicine, Monash University, Melbourne, Australia; Department of Gastroenterology, Alfred Health, Melbourne, Australia; School of Translational Medicine, Monash University, Melbourne, Australia; Department of Gastroenterology, Alfred Health, Melbourne, Australia; School of Translational Medicine, Monash University, Melbourne, Australia

**Keywords:** inflammatory bowel disease, disease activity assessment, intestinal ultrasound, colonoscopy

## Abstract

**Introduction:**

Endoscopy has been the gold standard for the assessment of inflammatory bowel disease (IBD) activity. Increasingly, intestinal ultrasound (IUS) is utilized as an alternative non-invasive disease monitoring strategy. The aim of this study was to quantify and compare endoscopy usage for evaluation of IBD disease activity before and after the introduction of an IUS service.

**Methods:**

A retrospective single-center study was performed. Total numbers of lower gastrointestinal endoscopies performed for assessment of disease activity in luminal Crohn’s disease or ulcerative colitis were collected across two 5-year time periods: the pre-IUS era (2010-14) and the IUS era (2015-19). Endoscopies for dysplasia surveillance were excluded. The primary outcome was a comparison between the pre-IUS and IUS eras of the number of endoscopies performed for IBD activity assessment annually relative to the number of annual patients reviewed.

**Results:**

The number of endoscopies performed for IBD activity assessment decreased from 576 in the pre-IUS era to 474 in the IUS era despite an increase in cumulative annual patients (1746 vs 3080 patients in the pre-IUS and IUS eras, respectively). The proportion of cumulative annual endoscopies relative to cumulative patients reduced from 33 per 100 patients (pre-IUS era) to 15 per 100 patients (IUS-era) (incidence rate ratio 0.47, 95% CI 0.41-0.53; *P* < .001), a reduction of 53%.

**Discussion:**

Following the introduction of an IUS service, the number of endoscopies performed for evaluation of IBD activity was halved. The potential workflow and cost savings of this reduction in endoscopy utilization may be significant.

## Graphical abstract

**Graphical Abstract:**
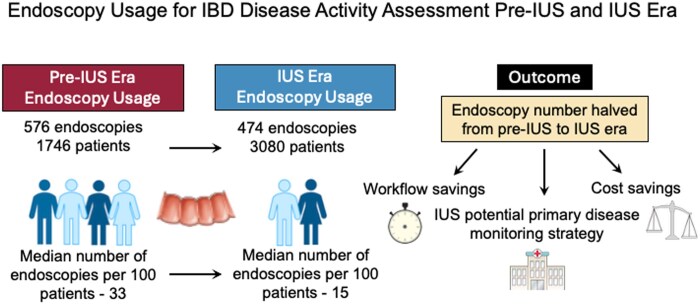

## 1. Introduction

Inflammatory bowel disease (IBD) is a chronic immune-mediated inflammatory disease with rising global incidence and prevalence.^1^^,^^2^ Despite significant advances in medical therapies in recent decades, IBD remains incurable. In addition to symptom control, treatment goals, achieved by a treat to target strategy, are the induction and maintenance of endoscopic remission, which is associated with improvements in quality of life and reductions in hospitalizations and surgeries.^3^

Endoscopy is the primary disease activity assessment and monitoring strategy in IBD but has practical challenges including accessibility, cost, risk, and patient acceptance.^4–7^ Non-invasive biomarkers such as C-reactive protein (CRP) and fecal calprotectin, although endorsed as formal treatment targets by the STRIDE II guidelines, have limitations including high test–test variability, lack of gut-specificity for CRP and variable accessibility and reimbursement for fecal calprotectin.^3^^,^^8^ Alternative imaging strategies in Crohn’s disease (CD), such as computed tomography (CT) and magnetic resonance imaging (MRI) have their own limitations, including radiation exposure and variable accessibility, respectively, and cannot be performed as point of care investigations in the clinic.

Intestinal ultrasound (IUS) has rapidly emerged in the last decade as a highly accurate and practical alternative non-invasive disease monitoring strategy in IBD. IUS assesses objective markers of intestinal inflammation including bowel wall thickness, hyperemia and stratification, mesenteric hypertrophy, and associated lymphadenopathy.^9–14^ IUS is accurate in distinguishing inflammatory versus non-inflammatory conditions when compared to the gold standard of ileocolonoscopy.^15–17^ In CD, systematic reviews and meta-analyses show IUS has a sensitivity and specificity of 84%-89% and 86%-94%, respectively. In CD, use of IUS in serial disease monitoring is accurate in assessing both small bowel and colonic CD, including response to medical therapy.^18–20^ IUS is also accurate in identifying transmural CD complications such as strictures, fistulas, and abscesses.^18^^,^^21^^,^^22^ More recently IUS has been shown to also be accurate in monitoring disease activity and response to medical therapy in ulcerative colitis (UC), although rectal views can be suboptimal.^13^^,^^23^^,^^24^ IUS also offers several practical advantages over other disease monitoring strategies. It is safe and non-invasive, with no radiation exposure nor need for contrast in most cases.^13^^,^^14^ There is no need for fasting, bowel preparation, or sedation, making patient acceptance high.^25^ As a point of care test that is easily repeatable, IUS can be utilized to facilitate real-time treatment decisions during a clinic consultation.^14^^,^^15^^,^^26^

IUS appears to be cost effective relative to MRI and endoscopic IBD assessment.^27^^,^^28^ More recent data show that utilization of IUS may decrease endoscopy usage in IBD. In a single-center Indian study, IUS use helped avoid colonoscopy in 51.4% of patients with CD and 51.8% with UC.^29^ Accordingly, in this study we aimed to quantify and compare endoscopy usage for evaluation of IBD disease activity before and after the introduction of an IUS service. We hypothesized that since the introduction of IUS, endoscopy usage for evaluation of IBD disease activity has reduced significantly.

## 2. Methods

A retrospective, single-center study was performed comparing the number of endoscopies performed for the evaluation of IBD disease activity in two pre-defined 5-year periods, the pre-IUS era (January 1, 2010 to December 31, 2014) and the IUS era (January 1, 2015 to December 31, 2019). Total numbers of lower gastrointestinal endoscopies (ileocolonoscopy or flexible sigmoidoscopy) performed for luminal CD or UC disease activity evaluation were recorded during these two time periods. From the IUS era, the number of IUS performed to evaluate IBD disease activity in CD and UC were also recorded. At our institution IUS is performed by accredited sonologists, either at dedicated IUS outpatient lists or during clinic visits within the twice weekly IBD outpatient clinics. Referrals for IUS are from clinic consultants who can refer for any of IUS, fecal calprotectin, or endoscopy.

The primary outcome was defined as a comparison of the cumulative number of endoscopies (ileocolonoscopy or flexible sigmoidoscopy) performed for IBD activity evaluation annually relative to the annual number of patients reviewed in the IBD clinic in the pre-IUS and IUS eras. Correction for patient numbers seen annually was necessary as the number of patients reviewed in the clinic increased significantly throughout the total 10-year study period. This outcome was calculated from the total endoscopies performed and total patients seen over each 5-year study period, rather than annual comparisons of individual years within the study period. Secondary outcomes included the total, yearly, and per-patient number of endoscopies in the pre-IUS and IUS eras, and the number of IUS performed on a per-patient, yearly and 5-yearly basis in the IUS era.

The endoscopy database (Endobase^®^) was queried with search terms including “inflammatory bowel disease, IBD, Crohn’s disease, ulcerative colitis” to generate a list of the total number of endoscopies performed during the two 5-year periods. The generated list was manually screened to identify IBD patients who underwent endoscopies for IBD disease activity evaluation. This included, but was not limited to, a change of clinical symptoms, abnormal biochemical, or radiological investigation results, and recent medication changes for disease flares. The IBD subtype of either CD or UC was recorded for each endoscopy performed. Patients with inflammatory bowel disease unclassified (IBD-U) were categorized as UC. Patients were excluded if endoscopies were performed for dysplasia surveillance, for evaluation of peri-anal CD, and for investigation of pouchitis.

The IUS database is a prospectively maintained database of all IUS scans performed within our department. The IUS database was queried and all patients who had IUS performed within the two pre-defined time periods were included. IUS reports were then manually assessed, and the number of IUS performed and whether they were performed to identify active disease or disease in remission was recorded. This study was approved by the Alfred Health Research Ethics Committee (Project 173/25). All authors had access to the study data and reviewed and approved the final manuscript.

**Figure 1.**
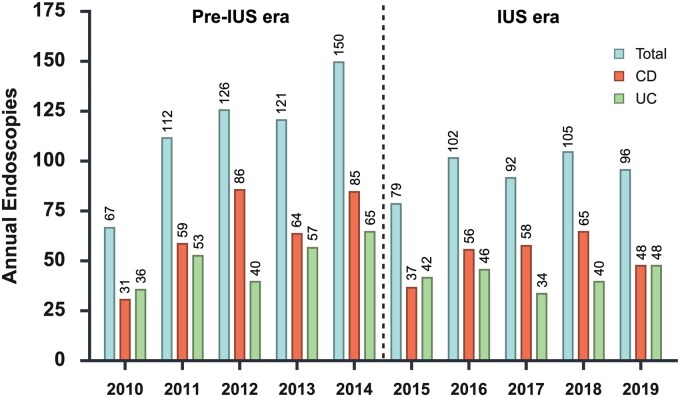
Total number of endoscopies (colonoscopy or flexible sigmoidoscopy) in each year in the pre-IUS and IUS eras. IUS, intestinal ultrasound; CD, Crohn’s disease; UC, ulcerative colitis.

For descriptive statistics, continuous variables are presented as median and interquartile ranges or means and standard deviations according to their distribution. Continuous variables were compared using the Student’s *t*-test or Mann–Whitney *U*-test, as appropriate. Categorical variables are presented as numbers (frequency) and percentages and were compared using chi-square tests. Endoscopy utilization was analyzed as a rate using Poisson regression with a log link function. The annual number of endoscopies performed for assessment of IBD disease activity was modeled as the outcome, with the annual number of patients reviewed included as an offset to account for differences in patient volume between eras. Robust standard errors were used to account for potential overdispersion. Results are presented as incidence rate ratios (IRRs) with 95% confidence intervals. A *P* value of <.05 was considered statistically significant. Statistical analysis was performed using Graphpad Prism v.10.6.0. Figures 1 and 4 were edited using BioRender (https://Biorender.com).

## 3. Results

**Figure 2.**
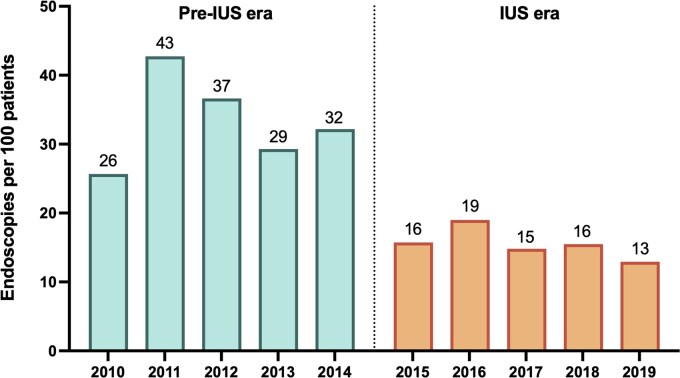
Annual number of endoscopies to assess disease activity per 100 patients in the pre-IUS and IUS eras. IUS, intestinal ultrasound.

In the pre-IUS era a total of 576 endoscopies were performed for IBD activity evaluation (325 for CD and 251 for UC). In the IUS era a total of 474 endoscopies were performed for IBD activity evaluation (264 for CD and 210 for UC). This reduction occurred despite an increase in the number of total annual patients reviewed, from 1746 in the pre-IUS era to 3080 in the IUS era. Figure 1 shows the year-by-year total number of endoscopies performed in the pre-IUS and IUS time periods. The number of endoscopies performed was then corrected for the number of patients reviewed, given the increase in patients seen over the duration of the two 5-year study periods. The number of endoscopies performed per 100 patients annually in the pre-IUS and IUS eras is shown in Figure 2.

After the introduction of the IUS service, the overall annual cumulative number of endoscopies per patient decreased from 33 per 100 patients in the pre-IUS era to 15 per 100 patients in the IUS era. In Poisson regression analysis, the rate of endoscopy was 53% lower in the IUS era compared with the pre-IUS era (IRR 0.47, 95% CI 0.41-0.53; *P* < .001).

**Figure 3.**
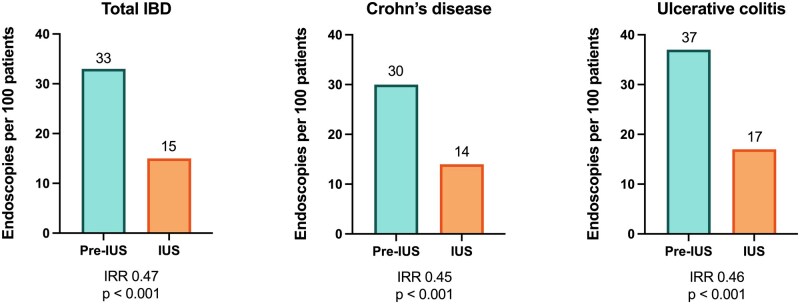
Number of endoscopies to assess disease activity per 100 patients in the pre-IUS and IUS eras according to disease subtype. IUS, intestinal ultrasound; IRR, incidence rate ratio.

In CD patients, the annual cumulative number of endoscopies per patient decreased from 30 per 100 patients in the pre-IUS era to 14 per 100 patients in the IUS era (IRR 0.45, 95% CI 0.38-0.53, *P* < .001). In UC patients, the annual cumulative number of endoscopies per patient review decreased from 37 per 100 patients in the pre-IUS era to 17 per 100 patients in the IUS era (IRR 0.46, 95% CI 0.38-0.55; *P* < .001), as is shown in Figure 3.

**Figure 4.**
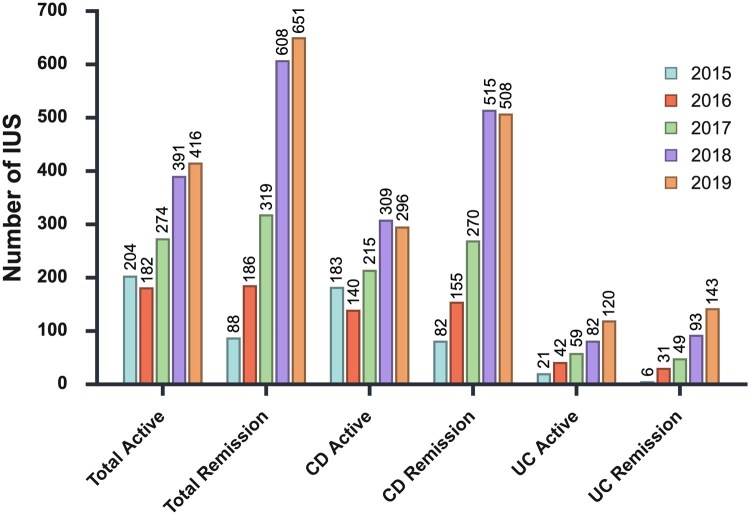
Total number of IUS scans done for both evaluation of active disease and routine monitoring in IBD patients in clinical remission. IUS, intestinal ultrasound; CD, Crohn’s disease; UC, ulcerative colitis.

During the 5-year IUS era a total of 3319 IUS were performed on IBD patients for either evaluation of active disease or routine disease monitoring in IBD patients in clinical remission. Of those, 1467 IUS were performed for the evaluation of active disease (44 per 100 patients per year), 1143 for CD and 324 for UC. In total, 1852 IUS were performed for objective confirmation of sonographic remission in patients in clinical remission (55 per 100 patients per year), 1530 for CD and 322 for UC. Figure 4 shows the annual numbers of IUS performed by diagnosis and indication during the IUS era.

## 4. Discussion

IUS has become an increasingly utilized alternative disease monitoring strategy in the treat-to-target era in IBD given its accuracy, safety, non-invasiveness, cost-effectiveness, and reproducibility.^14^ The SCOPELESS study aimed to quantify the reduction in endoscopies that has occurred due to the introduction of an IUS service. We have demonstrated that the number of endoscopies performed for evaluation of IBD disease activity has halved, with a similar magnitude of reduction seen in both CD and UC. Per-patient analyses confirmed that this reduction in endoscopies occurred despite an increase in the number of patients seen in our IBD clinic over the duration of the study period. This reduction in the use of endoscopy for IBD disease activity assessment may help to reduce endoscopy waiting lists by redirecting endoscopy use to other patient groups.

We also identified a consistent increase in the number of IUS performed each year during the 5-year IUS era, with over 3000 IUS scans performed in total. IUS was performed in both CD and UC and across all disease phenotypes and severities. Over half of the scans were performed in patients in clinical remission, in whom IUS was used as a complementary non-invasive disease monitoring tool to fecal calprotectin.

The strengths of our study include the large sample size of both endoscopies and IUS scans and the long study period of 10 years. Accurate data collection was facilitated by the use of two prospectively maintained institutional databases. Our study also has several limitations. It is a single-center retrospective study of an IBD tertiary referral center, and therefore prone to referral and selection bias. Our center is also highly experienced in the use of IUS, meaning these results may not be widely applicable to all clinical settings where IUS is less commonly used or available. To achieve comparable outcomes, recognized certification of sonographers would be required, as can be achieved via the GENIUS (Australia), IBUS (Europe) or iUSCAN (United States and Canada) credentialling programs.^30^^,^^31^ Similarly, given endoscopies are not performed on a fee for service basis at our institution there is no financial disincentive to clinicians in referring for an IUS rather than a colonoscopy. These remunerative processes may not be applicable in all practice settings. Patients were included only by diagnosis of CD and UC, without further demographic classification and analysis by disease phenotype, severity, or treatment exposure, which would be of interest in future studies. Future studies would also ideally quantify and analyze IBD-U patients independently. Similarly, we did not record the number of patients excluded from the initial Endobase search due to non-disease activity assessment endoscopy indications (dysplasia surveillance, perianal CD, pouchitis), potentially introducing selection bias. A minor limitation is that our analysis compared era-level endoscopy utilization to measure differences in healthcare utilization and did not account for temporal autocorrelation or clustering due to repeated endoscopies within individuals. However, while some patients did undergo multiple procedures, this would have occurred in both eras. Given the magnitude of the observed reduction in endoscopy usage in the post-IUS era, it is unlikely that adjustment for within-patient correlation would substantially alter the findings. Similarly, we compared two aggregated 5-year time periods rather than comparing annual temporal trends via an interrupted time series analysis, which may add further granularity to our results. However, the annual data presented in Figure 2 suggest an immediate and sustained reduction in endoscopy utilization after the introduction of IUS, rather than a simple continuation of a pre-existing temporal trend. The timing of the two 5-year study periods intentionally excluded service disruptions from the COVID-19 pandemic, which affected both IUS and endoscopic services from March 2020. Given the continued growth of the IUS service following the pandemic, it is likely that we would see an even greater reduction in the use of endoscopies if more recent years could have been used to define the IUS era. We acknowledge also that the timing of the two study periods coincided with the gradual adoption of the treat-to-target strategy during which the use of non-invasive tight disease monitoring increased, which itself may have influenced the frequency of patient reviews. This increase in the denominator of patient reviews may have led to an over-estimation of the magnitude of the reduction in colonoscopy use, although we believe this effect would be minor. Similarly, and importantly, we did not record how use of other non-invasive disease monitoring strategies, in particular the measurement of fecal calprotectin, changed throughout the study period, which may also have contributed to reduced endoscopy usage. We acknowledge that this limitation significantly reduces the causal inference we can make between intestinal ultrasound usage and endoscopy usage in our cohort. During the study period fecal calprotectin was only performed by external private pathology providers, meaning that data quantifying calprotectin measurements by the study cohort were not obtainable for analysis. We also did not record whether the use of IUS, instead of colonoscopy, for the assessment of disease activity was associated with equivalent or even better clinical outcomes for patients. From this cohort we plan to report rates of clinical and endoscopic remission, hospitalizations, surgeries, and the need for treatment-escalation in IUS-monitored and endoscopy-monitored patients in a future analysis. This analysis, and other ideally prospective studies, will be important to help determine whether there are potential negative outcomes associated with the introduction of an IUS service. Whether treating to a target of transmural healing, as assessed by IUS, is superior to a target of clinical and biomarker remission in improving clinical outcomes including endoscopic remission is the subject of the ongoing VECTORS study.^32^

The SCOPELESS study has found that in the 5 years following the introduction of an IUS service, the number of endoscopies performed for IBD disease activity evaluation was halved. While the causality of this association cannot be proven from our data, with IUS being performed for both assessment of disease activity and objective confirmation of clinical remission, the potential workflow and cost savings of reducing endoscopies for IBD disease activity may be significant. IUS has the potential to become the primary disease monitoring strategy in IBD.

## Data Availability

The study data may be shared with researchers who submit a research proposal approved by the Alfred Health Human Research Ethics Committee and at the discretion of the corresponding author. If approval is granted, deidentified and anonymized datasets will be shared. Requestors will be required to sign a data access agreement. Proposals should be sent to the corresponding author at m.sparrow@alfred.org.au.
